# Congenital oropharyngeal teratoma in a neonatal goat and brief review of extragonadal teratomas in animals

**DOI:** 10.1177/10406387251410495

**Published:** 2026-01-29

**Authors:** Sang-Joon Lee, Pedro Alejandro Triana Garcia, Eunju April Choi

**Affiliations:** Keyprime Research, Cheongju-si, Chungcheongbuk-do, South Korea; Veterinary Medical Teaching Hospital, School of Veterinary Medicine, University of California–Davis, Davis, CA, USA; Department of Pathology, Microbiology and Immunology, School of Veterinary Medicine, University of California–Davis, Davis, CA, USA

**Keywords:** congenital, extragonadal, goats, oropharyngeal, teratoma

## Abstract

Teratomas originate from pluripotent germ cells and differentiate into the 3 germ cell layers: endoderm, mesoderm, and ectoderm. Hence, these tumors arise most often in the gonads. Extragonadal teratomas are rare in veterinary medicine. Congenital oropharyngeal teratoma, also known as epignathus, is a neoplasm that has been reported in humans and a few veterinary species. We describe the clinical, gross, cytologic, and histopathologic features of an oropharyngeal teratoma in a neonatal Boer × Nigerian Dwarf goat that died within 4 h of birth, and briefly review extragonadal teratomas in veterinary species.

Congenital neoplasms are classified as neoplastic lesions that are detectable at birth or within the first 2 mo of life.^26^ They are still considered congenital even if identified up to 2 mo into the postnatal period, provided their origin can be traced back to embryonic development.^26^ Although rare, a wide range of congenital neoplasms have been sporadically reported across domestic species, including pigs,^27^ horses,^28^ cattle,^18,25,26,46^ dogs,^43^ and cats.^42^

Congenital neoplasms are considered rare in small ruminants.^44^ Our search of PubMed using search terms “goat” OR “sheep” OR “small ruminant” AND “congenital” AND “tumor” yielded 11 results, of which 1 matched the criteria. The same search in Google identified 4 additional cases of congenital neoplasms, confirming that this condition is rarely reported. The 5 cases identified included a fetal rhabdomyoma in a stillborn Ardi goat kid,^2^ an intracranial teratoma in a 1-d-old lamb,^34^ a cutaneous hemangioma in a 5-d-old lamb,^3^ an intradural melanoma in a 9-d-old Saanen goat kid,^44^ and an extraneural hemangioblastoma in a 1-mo-old lamb.^51^

A 2.54-kg, 4-h-old, female Boer × Nigerian dwarf kid was noted by the owner to be weak and non-suckling since birth. On presentation to the University of California, Davis, Veterinary Medical Teaching Hospital (Davis, CA, USA), the lamb was dyspneic, had marked respiratory acidosis, and lacked the suckle reflex. Thoracic ultrasound revealed aspiration pneumonia, particularly affecting the cranioventral lung fields. During attempts at intravenous catheter placement, the animal experienced cardiac arrest and could not be resuscitated. Laryngoscopy performed during cardiopulmonary resuscitation by the submitting clinicians identified a large, firm nodule located in the caudal soft palate, suspected to be obstructing the airway.

A routine autopsy was performed. Grossly, an expansile, well-demarcated, 2 × 1.5 × 1-cm mass nearly completely filled and occluded the oropharynx and extended into the caudal aspect of the nasopharynx (**Figs. 1, 2**). The mass was partially attached to the pharyngeal mucosa, compressed the soft palate, and extended caudally to the epiglottis. On section, the mass was gray-to-tan, firm-to-hard, and gritty. Other findings included moderate, regionally extensive, bronchopneumonia of the right cranial lung, suspicious for aspiration pneumonia. The rumen, reticulum, and abomasum were filled with abundant milk, consistent with a failure of closure in the esophageal groove.

**Figures 1–6.**
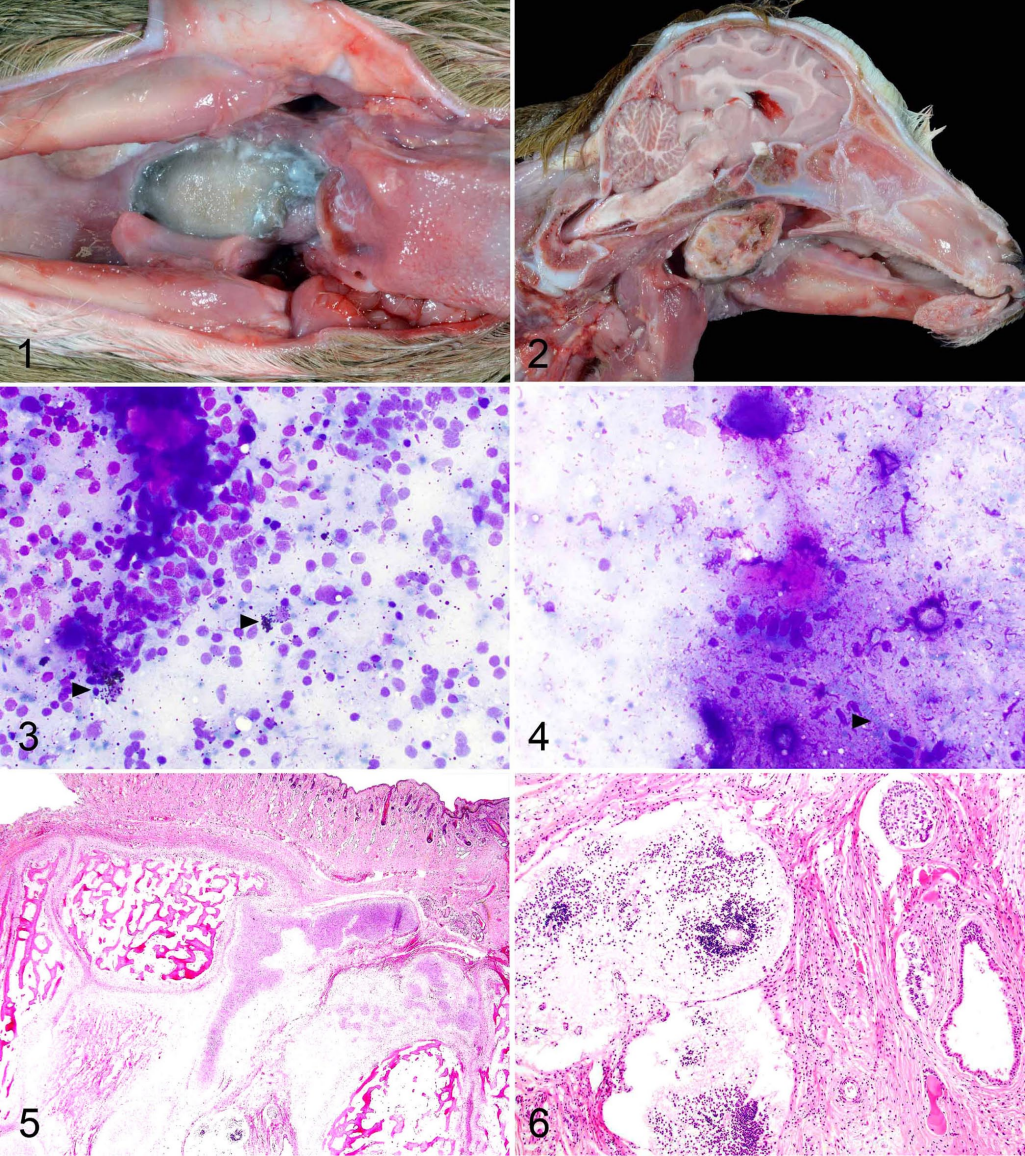
Gross, cytologic, and histologic features of a congenital oropharyngeal teratoma in a neonatal goat. **Figure 1.** The tongue is reflected caudally to the right to expose the sharply demarcated, tan-to-dark-gray, occlusive oropharyngeal mass at the midline. **Figure 2.** On midline section, the mass has variable texture and fills the oropharynx. **Figure 3.** Impression smear of cohesive cells (endoderm or ectoderm) and melanocytes (ectoderm; arrowhead). Diff-Quik. **Figure 4.** Impression smear highlighting a cohesive raft of cells associated with pink matrix (suspect endoderm) and striated spindle cells (mesoderm; arrowhead). Diff-Quik. **Figure 5.** Histologic features with external covering of well-differentiated pigmented haired skin and adnexa (ectoderm) with internal well-developed bone, cartilage, and collagen (mesoderm). H&E. **Figure 6.** Central to the mass is a focus of cells resembling neural tissue (ectoderm) to the left, and glandular structures suspected to be of endodermal lineage to the right. H&E.

A wide variety of cells exfoliated in impression smears of the mass (**Figs. 3, 4**). Identifiable components include pigmented melanocytes (ectoderm; Fig. 3), tubular epithelial structures (suspect endoderm; Fig. 4), keratinocytes (ectoderm), and skeletal muscle fibers (mesoderm; Fig. 4) embedded within a basophilic, fibrillar matrix.

Histologically, the periphery of the mass was covered circumferentially by haired skin with an epidermis and dermis that had loosely arranged collagen with numerous blood vessels, brown adipose tissue, hair follicles, and sebaceous and apocrine glands (**Fig. 5**). Deep to the dermal tissue was a somewhat organized plate of lamellar and woven bone separated by loose connective tissue (marrow cavity), hematopoietic cells, and several lobules of well-differentiated hyaline cartilage (Fig. 5). Some regions were expanded by myxomatous matrix or adipose tissue. There were lobules of fat and collagen (mesoderm), small blue cells resembling neural or neuroectodermal tissue (ectoderm), and cysts lined by epithelium or forming small lobule-like structures (suspect endoderm or ectoderm; **Fig. 6**). No indication of malignancy was noted. Moderate, localized aspiration pneumonia was confirmed with the presence of proteinaceous material (milk) and neutrophils in the airways. The cause of death was asphyxia resulting from an obstructive pharyngeal teratoma, specifically a mature teratoma.

Teratomas are congenital tumors that arise from pluripotent germ cells.^7^ At least 2 embryonic germ layers (ectoderm, mesoderm, endoderm) are present; teratomas therefore arise most often in the gonads (ovaries and testes).^7^ Extragonadal teratomas are rare in veterinary medicine. Cases of congenital oropharyngeal teratomas have been reported in humans,^36^ cats,^32,52^ a sable antelope,^12^ and potentially a calf.^1^ Congenital orofacial teratomas are also called epignathus and can rarely extend into the cranium in affected neonates.^21^ The cell(s) of origin for oropharyngeal teratomas is unknown. However, pluripotent cells in the notochord during early embryogenesis or in the region of Rathke pouch are hypothesized as potential candidates.^19,33^ Other congenital oral neoplasms affecting the gingiva and lip have been reported in domestic species (e.g., squamous cell carcinoma in a pig^13^ and hemangiosarcoma in a foal^9^). For a congenital oropharyngeal mass, teratoma should be considered a possibility.

A notable species in which extragonadal teratomas have been reported is the cat (9 of 41 cases, 22%; **Table 1**). Age at presentation was highly variable, ranging from immediately after birth to decades later (Table 1). Common locations include the head, especially the eye, the oropharyngeal region, and the retroperitoneum. Four ferrets were reported to have adrenal teratomas in a case series,^48^ and 2 case reports described coccygeal teratomas in young cats,^20,45^ but most cases were sporadic.

**Table 1.** Location, species, and age of animals diagnosed with extragonadal teratoma reported in the literature.

Anatomic location	Species	Age
Coccygeal region (tail)	Cat (*Felis catus*)^20^	6 mo
	Cat (*Felis catus*)^45^	8 mo
Head		
Base of left ear	Cat (*Felis catus*)^47^	4 mo
Orbital and third eyelid	Cat (*Felis catus*)^4^	9 wk
Retrobulbar	Cat (*Felis catus*)^50^	3 y
	Great blue heron (*Ardea herodias*)^40^	Adult
Retrobulbar, intracranial	Lesser kestrel (*Falco naumanni*)^24^	10 d
Extraocular	Domestic turkey (*Meleagris gallopavo domestica*)^37^	6 wk
Oropharyngeal	Cat (*Felis catus*)^32^	2 mo
	Cat (*Felis catus*)^52^	5 mo
	Cow (*Bos taurus*)^1^	2 d
	Sable antelope (*Hipptragus niger*)^12^	0 d
Right parietal bone/subcutis	Domestic duck (*Anas platyrhynchos domesticus*)^16^	10 mo
Intracranial		
Left pons	Domestic duck (*Anas platyrhynchos domesticus*)^15^	8 mo
Left ventral meninges	Alpaca (*Vicugna pacos*)^15^	4 y
Right colliculi	Sheep (*Ovis aries*)^34^	1 d
Right hemisphere	Fantail pigeon (*Columba livia domestica*)^17^	1 y
Suprasellar	Pampas deer (*Ozotoceros bezoarticus*)^14^	Young
	Rabbit (*Oryctolagus cuniculus*)^7^	Young
Thalamus	Cat (*Felis catus*)^8^	4 mo
Spinal cord, cervical	Dog (*Canis lupus familiaris*)^49^	11 y
Internal		
Cranial celom	Pekin duck (*Anas platyrhinchos domesticus*)^29^	7 mo
Mediastinal	American black bear (*Ursus americanus*)^30^	2.75 y
Merkel diverticulum	Chicken (*Gallus gallus domesticus*)^47^	4 wk
Adrenal gland	Cow (*Bos taurus*)^22^	Unk
	Domestic ferret (*Mustela putorius furo*)^48^	4 mo
	Domestic ferret (*Mustela putorius furo*)^48^	1 y
	Domestic ferret (*Mustela putorius furo*)^48^	3 y
	Domestic ferret (*Mustela putorius furo*)^48^	Adult
Retroperitoneum	Cynomolgus monkey (*Macaca fascicularis*)^41^	63 mo
	Rabbit (*Oryctolagus cuniculus*)^31^	4 mo
	Skunk (*Mephitis mephitis*)^29^	4 y
Urinary bladder	Maned wolf (*Chrysocyon brachyurus*)^10^	5.5 y
Subcutis, hindlimb, hock	Roe deer (*Capreolus capreolus*)^5^	Adult
Left perineal region	Cat (*Felis catus*)^35^	10 mo
Over keel	Red-crowned Amazon parrot (*Amazona viridigenalis*)^23^	42 y
Placenta	Horse (*Equus caballus*)^11^	0 d
Umbilical cord	Horse (*Equus caballus*)^6^	0 d
Pectoral fin	Endlers (*Poecilia wingei*)^38^	2 mo
	Endlers (*Poecilia wingei*)^38^	2 mo

In people, teratomas are the most common extragonadal germ cell tumor in the prepubertal age group, and common sites of origin include the sacrococcygeal, intracranial, mediastinal, head, and peritoneal regions,^39^ similar to descriptions in animals. Prognosis or age at presentation seems to be related to location and speed of growth. Most animals with an oropharyngeal teratoma were presented neonatally because of the obstructive nature of the mass. Similarly, animals with an intracranial teratoma were presented at a young age, apart from a 4-y-old alpaca with a teratoma that mostly expanded the meninges.^14^ The neonatal kid in our case survived for 4 h and received medical attention. If the mass had not completely obstructed the nasopharyngeal space, surgical excision may have been an option.
